# Sensitivity Analysis of Acoustic Emission Detection Using Fiber Bragg Gratings with Different Optical Fiber Diameters

**DOI:** 10.3390/s20226511

**Published:** 2020-11-14

**Authors:** Georgios Violakis, Tri Le-Quang, Sergey A. Shevchik, Kilian Wasmer

**Affiliations:** 1 Empa—Swiss Federal Laboratories for Materials Science and Technology, Laboratory for Advanced Materials Processing (LAMP), Feuerwerkerstrasse 39, 3602 Thun, Switzerland; violakisg@hmu.gr (G.V.); Quang.Le@empa.ch (T.L.-Q.); sergey.shevchik@empa.ch (S.A.S.); 2 Department of Electrical and Computer Engineering, Estavromenos Campus, HMU—Hellenic Mediterranean University, 71410 Heraklion, Greece

**Keywords:** acoustic emission detection, acoustic sensors, fiber Bragg gratings, optical fibers, wavelet decomposition

## Abstract

Acoustic Emission (AE) detection and, in particular, ultrasound detection are excellent tools for structural health monitoring or medical diagnosis. Despite the technological maturity of the well-received piezoelectric transducer, optical fiber AE detection sensors are attracting increasing attention due to their small size, and electromagnetic and chemical immunity as well as the broad frequency response of Fiber Bragg Grating (FBG) sensors in these fibers. Due to the merits of their small size, FBGs were inscribed in optical fibers with diameters of 50 and 80 μm in this work. The manufactured FBGs were used for the detection of reproducible acoustic waves using the edge filter detection method. The acquired acoustic signals were compared to the ones captured by a standard 125 μm-diameter optical fiber FBG. Result analysis was performed by utilizing fast Fourier and wavelet decompositions. Both analyses reveal a higher sensitivity and dynamic range for the 50 μm-diameter optical fiber, despite it being more prone to noise than the other two, due to non-standard splicing methods and mode field mismatch losses. Consequently, the use of smaller-diameter optical fibers for AE detection is favorable for both the sensor sensitivity as well as physical footprint.

## 1. Introduction

Acoustic Emission (AE) detection is a valuable technique with proven performance in both non-destructive Structural Health Monitoring (SHM) [1] and medical diagnostics [2]. At present, the industry standard for AE detection is considered to be the piezo-electric transducer. However, in recent years, optical fibers have attracted considerable attention from researchers and industry alike, as an alternative with numerous successful examples [3,4]. Optical fibers offer an attractive platform for sensor deployment due to their small size (<150 μm) and weight, low cost, electromagnetic interference immunity, and high temperature stability, and in the case of AE detection, they can also provide a frequency response spanning from a few hertz up to tens of megahertz, accompanied by high sensitivity or a high dynamic range as stated and summarized in several review papers [3,5,6,7]. Furthermore, optical fibers are also exploited as acoustically controlled optical devices, such as notch filters [8].

AE detection through optical fibers has been mainly realized using two types of devices: optical fiber interferometers and Fiber Bragg Grating (FBG) sensors [5]. Optical fiber interferometers date back to 1977 [9], and there has been considerable work in the literature concerning their design and sensitivity optimization, with the most successful example of such a sensor being the Fabry–Perot cavity [10]. However, the majority of interferometric optical fiber AE detectors suffer from cross-sensitivity, due to the fact that the incoming acoustic signal information is amplitude encoded and is prone to alteration by factors such as cable bending, tension, temperature changes, etc. taking place far away from the acoustic source. Fabry–Perot cavities, on the other hand, overcome this shortcoming but are lacking in dynamic range, multiplexing capability and ease of handling.

The most successful implementation of an optical fiber AE detection sensor has been through a Fiber Bragg Grating [11]. FBGs for AE detection were first proposed in 1996 [12], and since then, numerous variations of both the FBG design as well as the sensing setup have been examined to maximize the capabilities of the sensor. Important milestones in FBG AE detection technology have been the introduction of *π*-shifted FBGs, which have been shown to extend the acoustic sensing range of optical fibers beyond tens of megahertz [13], as well as advanced sensing techniques [14], which cancel out noise and increase the dynamic range of the sensor. The implementation of novel optical fiber designs has also contributed to increased ultrasonic sensitivities as shown by Yang et al. [15]. Moreover, researchers have also examined a shift from fused silica optical fibers to novel, less stiff materials that could potentially provide higher sensitivities in the ultrasonic range. Acoustic emission detection sensors have been fabricated in polymethylmethacrylate (PMMA) [16,17,18] and CYTOP^®^ [19] optical fibers, which have demonstrated improved sensing capabilities, especially in the ultrasonic acoustic range. Specifically, FBGs [20] and interferometric sensing modalities [16] have been used in PMMA microstructured optical fibers, with reported frequency responses up to 3.5 and 10 MHz for the former and the later, respectively. Moving from PMMA to CYTOP^®^ optical fibers, recent publications report the successful detection of ultrasonic frequencies up to 15 MHz [19,21]. In general, the acoustic sensitivity of polymer optical fibers is at least one order of magnitude higher than that of silica optical fibers [4]. In addition, towards the direction of exploring novel acoustic emission detection-enhancing materials, there have been efforts in altering the external coating of fused silica optical fibers in order to improve ultrasonic detection sensitivity [22] as well as novel optical fiber holder designs in order to improve omni-directional sensitivity [23].

The effect of the optical fiber diameter on the sensitivity of optical fiber AE detection has been studied in the literature, mainly in the context of tapered optical fibers or fibers where the cladding was removed either mechanically or chemically. For instance, tapered optical fibers were used for AE detection in Li et al. [24], but these were without FBG sensors on them and required special handling and mounting, making them impractical for use outside the laboratory. Another approach undertaken to reduce the optical fiber diameter was the grinding of the fiber cladding, resulting in D-shaped optical fibers that were later exposed to laser irradiation to inscribe FBGs [25]. This approach, again, resulted in a fragile detector head, making it less appealing for practical-use-case scenarios. Finally, the optical fiber diameter has been reduced by means of etching with Hydrogen Fluoride (HF), a time-consuming and hazardous procedure [26]. Notwithstanding the non-practical nature of the previous processes, clear advantages in AE detection sensitivity have been reported for the miniaturized sensors.

In the present work, FBGs were inscribed in commercially available photosensitive optical fibers of 50 and 80 μm cladding diameters and tested for AE detection, while comparing performance to a commercially fabricated FBG (in 125 μm-diameter optical fibers) with similar spectral characteristics. The results show an advantage of the 50 μm optical fiber in detecting higher ultrasonic frequencies than the larger-diameter optical fibers, as well as a higher sensitivity throughout the whole ultrasonic range, under the given testing conditions. The direct inscription of FBGs in commercially available optical fibers is a convenient means of utilizing the benefits of smaller-diameter optical fibers in AE detection without resorting to complex fiber diameter reduction methods.

## 2. Materials and Methods

Fiber Bragg gratings were fabricated in-house in a commercially available photosensitive optical fiber, namely, SM1500, manufactured by Fibercore Ltd. Two different optical fiber diameters were used: 50 and 80 μm. Additionally, commercial FBGs in 125 μm-diameter optical fibers with similar spectral characteristics were purchased from Femto Fiber Tec GmbH and used for comparison. In order to interrogate the fabricated 50 and 80 μm-fiber FBGs, the optical fibers were fusion spliced to normal SMF28 optical fiber pigtails using a custom splicing parameter set. Spectral interrogation was performed through a Micron Optics sm125 FBG interrogator with a spectral resolution of 1 pm. The inscription of FBGs in the photosensitive optical fiber core took place using the standard phase mask technique. The illumination wavelength was 193 nm from an ArF excimer laser, and the phase mask pitch was 1060.00 nm. Typically, the irradiation time did not exceed 7 min at a repetition rate of 20 Hz and an energy per pulse of ~ 100 mJ/cm^2^. The laser temporal pulse width was 38 ns at full width half maximum (FWHM). It is worth mentioning at this point that in Plastic Optical Fibers (POFs), using a KrF laser with similar temporal characteristics leads to FBG inscription times less than 25 s for a variety of polymers used in POFs, such as PMMA, TOPAS, Polycarbonate (PC), or Zeonex [27].

**Figure 1:**
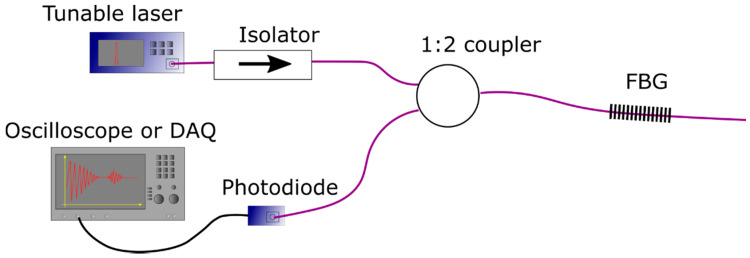
Acoustic emission detection setup based on the edge filter detection method. FBG: Fiber Bragg Grating.

Following the FBG fabrication, the sensing optical fibers were transferred to an AE detection setup, which was based on the edge filter detection method [28]. It consisted of a tunable laser with a 100 kHz linewidth (Yenista TLS WDM), which was set to operate spectrally within the linear slope of the FBG. The light was distributed to the sensing element (FBG) and to the photodiode detector (PD) through a 1:2 optical fiber coupler (50:50 optical power splitting between ends). Between the coupler and the laser, a > 40 dB isolator was used to prevent light back-reflections reaching the source. The PD response to the acoustic waves was recorded using a Lecroy model HDO6104 oscilloscope. A schematic representation of the AE setup is illustrated in Figure 1.

The setup was installed inside a temperature-controlled room, and all the measurements took place at a temperature of 25 °C and a relative humidity of 40–60%. It is well-known that FBGs exhibit strain–temperature cross-sensitivity [29]. Since the AE detection setup used in this work did not include any temperature compensation mechanism, care was taken to undertake all the measurements in an environment with minimal temperature variations, so that the acquired acoustic signals (derived from the strain variation exerted by the acoustic/vibrational shockwave) were not altered by temperature changes. The minimization or elimination of the effect of temperature has been reported in the literature using chirped and strained FBGs [30], tilted FBGs (cladding-core recoupling) [31] or FBGs in multicore optical fibers [32].

**Figure 2:**
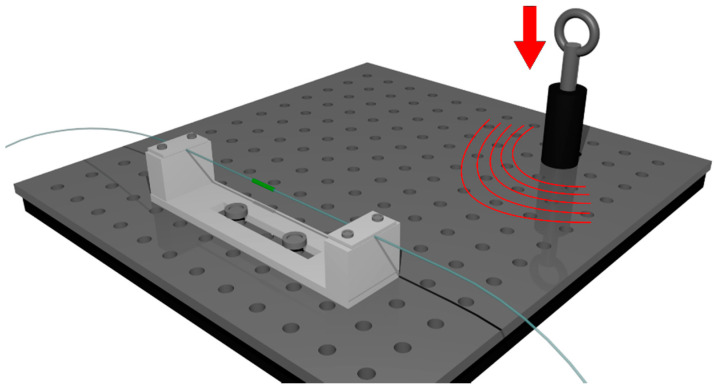
Acoustic emission generation setup: a stainless steel rod is raised at a fixed height in a fixed trajectory and released to produce an impact.

In order to compare the response of each sensor to the incoming acoustic wave, each optical fiber tested was placed in a U-shaped PMMA holder, with the fibers clamped at a distance of L ≈ 6.2 mm from either side of the FBG, which was placed in the middle. The optical fiber mount was clamped on a metallic surface of an optical table with an orientation similar to that shown in Figure 2. An adjustable height stainless steel rod along with its base plate was placed at a distance of 260 mm from the FBG. Acoustic waves were produced by lifting the rod to a specific height (h = 50 mm) and releasing it free to impact the base plate. This technique produced similar acoustic waves incoming to the FBG holder. A metallic solid impact on a massive body has been extensively used in the literature as a source of stress/acoustic waves [33,34], and it is often preferred to the ASTM standard pencil lead break test [35]. In this work, at least 6 drops of the rod were realized for each tested FBG, and a Fast Fourier Transformation (FFT) of the acquired signals revealed excellent overlap of the detected acoustic frequencies across the different tests, with amplitude variations of less than 9%.

## 3. Results and Discussion

### 3.1. FBG Fabrication and Calibrated Reflection Spectrum

**Figure 3:**
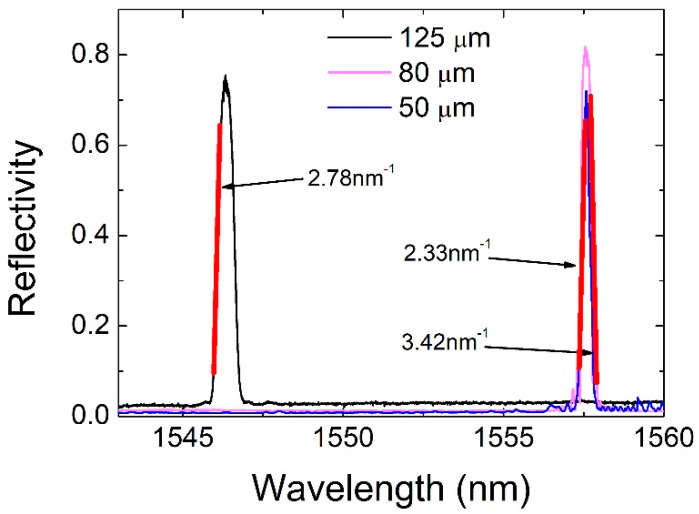
Calibrated reflection spectra of the FBGs inscribed in 125, 80 and 50 μm-diameter optical fibers.

The commercial FBG in the 125 μm optical fiber was fabricated using an apodized laser beam, resulting in a side-lobe-suppressed FBG, which typically provides a higher dynamic range for AE detection. In order to keep the sensitivity of all three sensors similar, the in-house-produced FBGs were fabricated with a slight chirp to reduce the side lobes and adjust the slopes to similar values. In this way, the three FBGs exhibited similar dynamic ranges and sensitivity. The calibrated reflection spectrum of all three FBGs is presented in Figure 3.

The in-house-fabricated FBGs overlapped spectrally, as they were manufactured using the same phase mask and similar inscription conditions. The bold red lines in Figure 3 are the slopes representing the linear response FBG range, with the arrows next to them denoting the calculated slope value. The linear response FBG range is defined as the spectral range either side of an FBG spectrum for which a linear fit can be performed with a coefficient of determination better than 0.98 and that covers at least 50% of the maximum FBG reflectivity. The reflectivity for all three FBGs lies in the range of ~70% to ~80%. To preserve similar spectral responses, the FBGs were mounted to the U-shaped PMMA holder by applying the same amount of strain, regulated with magnetic weights used during the optical fiber mounting. The optical fiber was clamped on one side of the PMMA holder, which was placed in a vertical orientation. On the free hanging part of the optical fiber, magnet pairs of three different predetermined weights were placed in order to apply a similar amount of strain to the optical fibers with three different diameters (see Equation (1)). Then, the fiber was clamped on the other side of the holder, the magnets were removed, and the holder was positioned in a horizontal orientation as shown in Figure 2.

### 3.2. Raw Acoustic Signal and Fast Fourier Transformation

The optical fiber mounted on the U-shaped holder resembles the acoustic behavior of a rod attached to a spring-mass setup, an analogy that was used for modeling a similar system by Beadle et al. [36]. They showed that the longitudinal strain $\frac{\partial \omega \left(x,t\right)}{\partial x}$ excreted in the optical fiber is inversely proportional to the optical fiber cross-section (see Equation (1) in Beadle et al. [36]):

(1) $$\frac{\partial \omega \left(x,t\right)}{\partial x}\propto \frac{F}{\rho AL^{2}}$$

where *F* is the acoustic radiation force, and *ρ*, *A* and *L* are the optical fiber density, cross-section and clamped length, respectively. Evidently, as the optical fiber diameter decreases, the applied strain increases, enhancing the sensor sensitivity.

**Figure 4:**
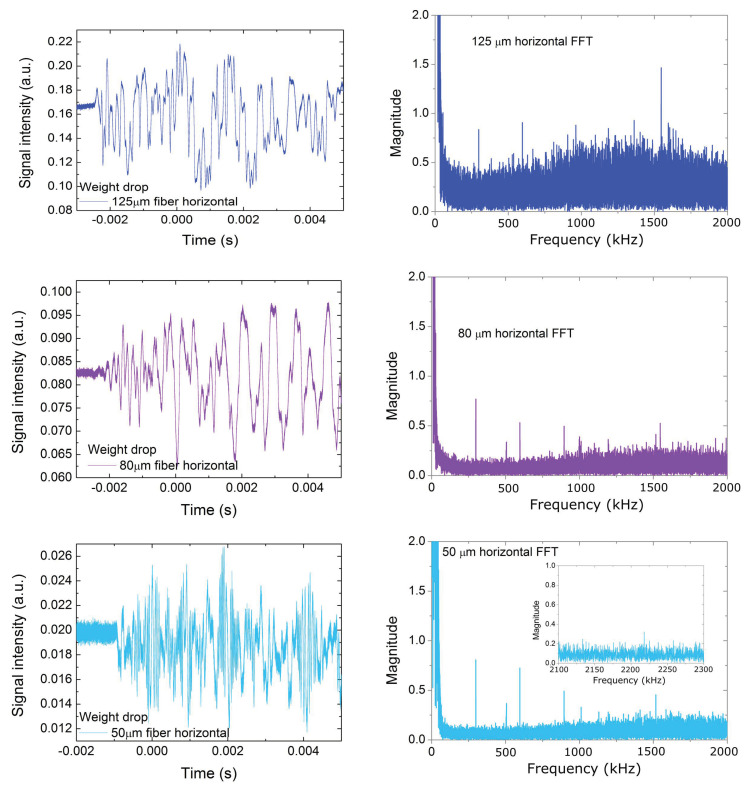
Left column: acoustic waveforms acquired from (top to bottom) 125, 80 and 50 μm-diameter optical fibers with similar FBGs. Right column: fast Fourier transformation of the waveforms in the right column, focusing on frequencies above 100 kHz.

The waveforms acquired after the impact of the rod for the three optical fiber diameters are presented in the left-hand column of Figure 4. The acquired waveforms were the sum of both airborne and structural shockwaves originating from the rod impact to the massive body. As the optical fiber surface was very small, the main source of the acquired signal was structural shockwaves, and, in particular, it was the transfer function of the PMMA optical fiber holder that coupled the incoming shockwaves to the optical fiber. As all the tests were undertaken under the same conditions, this transfer function was considered to be the same for all three different diameter optical fibers, and hence, the experimental results between them are comparable.

Based on Figure 4, two main features are readily observed in the waveforms. First, the spectral density of the 50 μm optical fiber is higher as compared to the other two. The acoustic impedance of a cylindrical rod can be estimated using [37]:

(2) $$Z_{f}=2c_{f}\rho_{f}A_{f}$$

where *c_f_* is the speed of the acoustic waves excited by the holder vibration, *ρ_f_* is the optical fiber density and *A_f_* is its cross-sectional area. Therefore, due to the direct proportionality of the acoustic impendence to the optical fiber cross-section, the smallest fiber of the group (50 μm) was expected to have an impendence that is 84% lower than that of the 125 μm-diameter fiber, resulting in higher sensitivities across the whole acoustic spectrum.

Second, the amplitude of the signal was stronger for the larger fiber and decreased with the fiber diameter. The reasons for this behavior are twofold. First, the optical fibers were spliced to standard telecommunications optical fibers. The smaller optical fiber cores had sizes and compositions different to those of the standard optical fiber. As a result, there was a difference in the Mode Field Diameter (MFD) between both fibers, resulting in mode field mismatch losses. Such losses can be as high as ~40% for two optical fiber splices with a mode field mismatch in the order of 20% [38] and can only be overcome by employing optical fibers of similar MFDs or mode field converters [39]. Second, apart from the mode field mismatch losses, the pronounced splice losses must be taken into account due to the use of an unoptimized non-standard splicing program for fusing together the optical fibers of standard and smaller diameters.

In the right column of Figure 4, a fast Fourier transformation of the acquired waveforms is presented, with emphasis on the ultrasonic frequencies. Both the FFT frequency plot range and magnitude are kept constant in all three graphs for the easier visualization of the differences. In addition, an inset for the 50 μm optical fiber is placed, showing signal detection at frequencies above 2 MHz, which was not detected by the larger-diameter optical fibers using our experimental setup.

The observation of the FFT graphs also shows a clearly higher noise level for the 125 μm optical fiber in the whole range > 100 kHz, as compared to the two smaller optical fiber diameters. This is evidenced by the higher mean signal value across the whole signal range above 100 kHz. A useful quantification of this noise level can be derived by calculating the signal-to-noise ratio (SNR) for some characteristic ultrasonic frequencies. The SNR was calculated using the following formula:

(3) $$SNR_{dB}=10log_{10}\left(\frac{P_{signal}}{P_{noise}}\right)$$

where *P_signal_* and *P_noise_* are the signal and noise levels of the acquired waveform FFT on a linear scale. *P_noise_* is calculated as the median amplitude value where no discrete signal can be identified. *P_signal_* is the amplitude of a frequency that exhibits at least a 20% higher amplitude than the noise level. *SNR_dB_* is the signal-to-noise ratio expressed in decibels (dB). The results are summarized in Table 1. The frequencies presented in this Table were also found to be excited when the optical fiber sensor was mounted vertically, i.e., with the optical fiber axis perpendicular to the metallic table, and are therefore considered to be representative of optical fiber FBG sensitivity irrespective of the optical fiber sensor orientation.

### 3.3. Wavelet Decomposition

Compared to their piezoelectric counterparts, FBG sensors have the notable advantage of detecting acoustic frequencies from a few hertz up to megahertz, i.e., over an extended detection range. Therefore, to compare the performance of the three optical fiber diameters, it is beneficial to use wavelet decomposition to visualize the sensitivity of the FBG sensor in different frequency ranges and obtain the time–frequency characteristics of the dynamic range. In this case, the characterization was carried out via discrete wavelet packet transform (DWPT) [40]. DWPT was specially developed to operate with non-stationary signals, thus having an advantage over classical Fourier decomposition [40], as it better suits the nature of the acoustic signals in this experiment.

**Figure 5:**
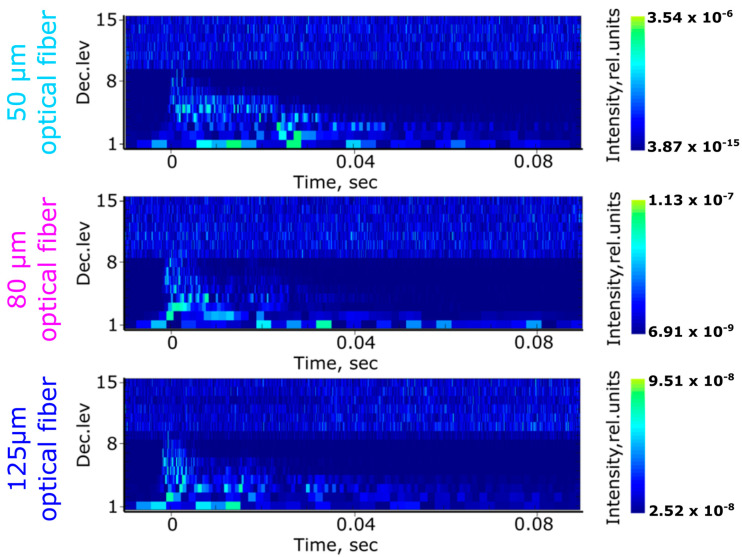
Spectrograms acquired using discrete wavelet packet transform for the three different-diameter optical fiber FBG sensors.

The idea behind wavelet transform is the recurrent decomposition of the signal according to the following terms (general wavelet formalism) [40]:

(4) $$\varphi_{j+1}\left(n\right)=\underset{r}{\sum }\sqrt{2}h_{0}\left(r\right)\varphi_{j}\left(2n-k\right),\;k\subset Z$$

(5) $$\psi_{j+1}\left(n\right)=\underset{r}{\sum }\sqrt{2}h_{1}\left(r\right)\varphi_{j}\left(2n-k\right)k\subset Z$$

where $\sqrt{2}$ is a scaling factor; *h*_0_ and *h*_1_ are the low- and high-pass wavelet filters, respectively; *r* is the filter length (e.g., the number of filter coefficients); *j* is the decomposition level (see Figure 5 *y*-axis); *n* is the specific time stamp of the sampling point or the wavelet coefficient in the time domain (i.e., see Figure 5 *x*-axis); and *k* is an offset. The output of Equations (4) and (5) is the low- and high-pass contents of the signals. In DWPT, these results are recurrently passed through Equations (4) and (5), while obtaining information about different frequency bands.

In this work, the relative energy from the frequency bands was used to characterize the sensor’s sensitivity to the acoustic stimuli. The relative energies were computed as:

(6) $$\rho_{normj,n}=\frac{E_{j}\left(n\right)}{E_{total}}$$

where $E_{j}\left(n\right)=\sum \psi_{j}{\left(n\right)}^{2}$ is the energy of a specific frequency band at scale *j* (see the *z*-axis in Figure 5), *n* is the time stamp as in Equations (4) and (5) and *E_total_* is the total energy accumulated over all the frequency bands. Under such circumstances, changes in the signal frequency and intensity read by the sensor are visualized at varying energy distributions across different frequency bands, providing a ”response map” as shown in previous work [41,42]. The resulting spectrograms of the three different-diameter optical fiber sensors derived from the wavelet decomposition are presented in Figure 5. The acoustic spectra used as input for the DWPT analysis are those already presented in Figure 4 (left column).

The intensity values in the spectrograms of Figure 5 are in relative units (see the color bar on the right of Figure 5). However, since the acoustic stimulus was similar for all three optical fiber diameters, we can compare the responses between the three sensors. The noise level of the acoustic signal is found in the spectrograms of Figure 5 before the time stamp 0 s, i.e., before the acoustic wave arrived at the sensor. The noise level mainly spanned 9–15 decomposition levels, while the acoustic signal lay between 0 and 9 decomposition levels. It is worth mentioning that the noise level remained similar before (time span < 0 s) and after (time span ≥ 0 s) the acoustic wave arrival.

A visual investigation of the spectrograms shows a better sensitivity of the 50 μm sensor to high frequencies, and evidence of this is visible in the larger number of frequency bands with elevated energies at higher decomposition levels (see the decomposition levels from 1 to 8). The 80 and 125 μm optical fiber sensors exhibited a lower density of states (detected frequencies) with lower energies (lower sensitivity). Additionally, the attenuation of the higher frequencies in the 50 μm sensor was lower as compared to that in the other two sensors, as already evidenced by the detection of the 2.2 MHz peak in the Fourier analysis.

The quantification of the information derived from the spectrograms clearly shows the performance differences between the three sensors. In particular, the response of the sensors depended on the optical fiber diameter and increased by several orders of magnitude for the smallest-diameter optical fiber as compared to the largest one (see the maximum intensity value color bar scale of Figure 5, where the noise levels 9–15 are not taken into account). The sensor’s response here was evaluated as the energy stored inside the wavelet frequency bands. The maximum sensor response values for the 125, 80 and 50 μm optical fiber FBGs were 9.51 × 10^-8^, 1.13 × 10^-7^ and 3.54 × 10^-6^, respectively. The dynamic range of the sensors was evaluated as the maximum and the minimum energy values stored in the wavelet frequency bands (again, excluding the noise level that was observed above the eighth decomposition level in the spectrograms in Figure 5). It was also observed that the dynamic range of the sensors differed significantly for the same acoustic distortion. Again, for the 125, 80 and 50 μm optical fiber FBGs, the dynamic range exhibited differences of 0, 2 and 9 orders of magnitude, as evidenced by the difference in the intensity level of the detected frequencies (see the difference between the maximum and minimum value in the *z*-axis of Figure 5, i.e., the color map).

## 4. Conclusions

In this work, a sensitivity analysis of FBGs in optical fibers with diameters of 125, 80 and 50 μm was performed for acoustic emission detection using a setup based on the edge filter detection method. The results show a significant advantage in using smaller optical fiber diameters for ultrasonic detection. The reason is that they offer both a higher sensitivity across the whole acoustic range and a higher dynamic range. This was demonstrated by the capability to detect ultrasound at higher frequencies than those detectable with larger optical fiber diameters. Fast Fourier transform and wavelet decomposition analyses were used to derive quantitative information on the sensors’ performance. It was found that both analyses agreed on the performance advantage of the 50 μm optical fiber in AE detection. However, wavelet decomposition provides additional advantages—in particular, the sensitivity and dynamic range mapping of the sensor over the full frequency response range. Hence, wavelet decomposition can be seen as a useful tool for both qualitative and quantitative sensor performance evaluation. A further performance increase in terms of the AE detection sensitivity of the 50 μm-diameter optical fiber can be obtained by improving the connection of such optical fibers to standard-diameter fibers, the reduction of mode field diameter mismatch losses and by optimizing the optical fiber holder design. A further improvement of the small-diameter optical fiber sensitivity is expected to be achieved by moving from silica optical fibers to polymer optical fibers, due to their inherently higher AE detection sensitivity.

## Figures and Tables

**Table 1:** Summary of signal-to-noise ratio values for different ultrasonic frequencies detected by the three optical fibers of different diameters.

Frequency (kHz)	Signal-to-Noise Ratio (SNR in dB) for Optical Fibers with Diameter:		
	125 μm	80 μm	50 μm
298.4	2.83	6.42	7.90
596.7	2.29	5.46	7.59
962.7	1.48	2.34	4.86
1546.6	3.20	3.40	3.45
2217.6	NA	NA	2.41
